# Development of a novel system based on single-tube nested real-time pcr system for the quantification of hazelnut in complex foods

**DOI:** 10.1186/2045-7022-3-S3-P144

**Published:** 2013-07-25

**Authors:** J Costa, MBPP Oliveira, I Mafra

**Affiliations:** 1 REQUIMTE, Department of Chemical Sciences, Faculty of Pharmacy, University of Porto, Porto, Portugal

## Background

Hazelnut is one of the most largely consumed tree nuts, which is used in a wide range of processed foods. However, it is classified as a potential allergenic ingredient and, consequently, should be declared in the label of pre-packaged foods (Directive 2007/68/EC), independently of its amounts. To verify the compliance with labelling and to safeguard the health of sensitised individuals, the development of new methodologies for the traceability of allergenic ingredients is essential [1].

## Methods

In this work, we propose the use of a novel approach based on single-tube nested real-time polymerase chain reaction (PCR) system to trace hazelnut in complex foods [2]. The recently developed system aimed gathering the advantages of nested PCR and real-time PCR technology. For the development and optimisation of this method for hazelnut detection, binary mixtures of wheat material spiked with hazelnut were prepared, ranging from 10% to 0.001%. Two sets of primers and a hydrolysis probe were designed targeting the gene encoding HSP1 protein for the specific detection and quantification of hazelnut.

## Results

The novel system evidenced high specificity and sensitivity, allowing a relative LOD of 50 mg/kg of hazelnut in wheat material, which is 2x lower than the LOD determined by the conventional real-time PCR system (100 mg/kg). It also allowed a 10-fold reduction of the absolute LOD to 0.5 pg of hazelnut (1 DNA copy). The method was successfully applied to processed foods (cereals, snacks, chocolates), highlighting its adequacy for the specific detection and quantification of hazelnut as potential hidden allergens in foods.

## Conclusion

In this work, an innovative and effective tool was proposed to trace minute amounts of hazelnut in foods. The method proved to be simple, specific, very sensitive and cost-effective, with potential for high-throughput DNA-based identification of hazelnut allergens in processed foods.

## Disclosure of interest

None declared.
